# First-trimester free thyroxine as an independent predictor of gestational diabetes mellitus: a retrospective cohort study

**DOI:** 10.1515/almed-2026-0007

**Published:** 2026-04-28

**Authors:** Thu T.T. Do, Thuy T. Le, Thu T.T. Ho, Lan T.N. Vu, Thuy C. Bui, Huong Thanh Hoang

**Affiliations:** Faculty of Medical Laboratory Science, Da Nang University of Medical Technology and Pharmacy, Da Nang, Vietnam; Da Nang Hospital for Women and Children, Da Nang, Vietnam; Faculty of Public Health, Da Nang University of Medical Technology and Pharmacy, Da Nang, Vietnam

**Keywords:** FT4, gestational diabetes mellitus, thyroid function, thyroid gland, TSH, pregnancy

## Abstract

**Objectives:**

Gestational diabetes mellitus (GDM) and thyroid dysfunction are two common endocrine diseases during pregnancy, both of which can adversely affect maternal and fetal health. Numerous studies have shown that abnormalities in thyroid hormones, particularly TSH and FT4, are associated with an increased risk of developing gestational diabetes. However, current research results are inconsistent due to differences in race, geographic location, and testing reference thresholds. This study aimed to evaluate the early predictive value of first-trimester thyroid stimulating hormone (TSH) and free thyroxine (FT4) levels for the development of GDM.

**Methods:**

A retrospective cohort study was conducted on 272 pregnant women from December 2023 to March 2025. Participants meeting inclusion criteria had TSH and FT4 levels measured in the first trimester (9–13 weeks) and underwent a 75 g oral glucose tolerance test between 24 and 28 weeks of gestation.

**Results:**

FT4 concentrations were significantly lower in the GDM group compared to the non-GDM group, whereas TSH levels showed no significant difference. An FT4 cut-off of ≤1.17 ng/dL predicted GDM with an area under the curve (AUC) of 0.715, a sensitivity of 86.5 %, and a specificity of 52.3 %. Multivariate logistic regression identified FT4≤1.17 ng/dL and pre-pregnancy body mass index BMI≥23 kg/m^2^ as independent risk factors associated with GDM.

**Conclusions:**

Low first-trimester FT4 levels are significantly associated with an increased risk of GDM. FT4 may serve as a valuable early predictive biomarker, suggesting a potential role for thyroid function screening in early pregnancy to optimize risk stratification.

## Introduction

Gestational diabetes mellitus (GDM) and thyroid dysfunction during pregnancy have been shown to have multiple adverse effects on pregnancy outcomes [1], [2], [3], with both conditions potentially causing short-term and long-term consequences for both mother and newborn. In addition to factors such as age, diet, BMI, and family history already known to influence GDM [1], [4], [5], [6], [7], [8], several studies have also shown an association between thyroid disorders and GDM [9], [10], [11], [12] and suggest that pregnant women with thyroid disorders should also be screened for GDM comprehensively.

According to the 2017 American Thyroid Association (ATA) guidelines, pregnant women should be screened for thyroid disorders at all stages of pregnancy, through evaluation of hormone levels such as thyroid stimulating hormone (TSH) and free tetraiodothyronine (FT4) [13]. Thyroid dysfunction is relatively common in pregnant women and is associated with several obstetric complications, including preterm birth and miscarriage, as well as adverse health outcomes in newborns [14], [15], [16]. In addition to documented pregnancy complications, there is growing evidence that imbalances of TSH and FT4 are involved in the pathogenesis of gestational diabetes [10], [11], [12, 17]. Numerous studies have shown that thyroid dysfunctions, whether latent or overt, can affect the development of gestational diabetes and vice versa [18], 19]. Therefore, understanding the relationship between gestational diabetes and thyroid function is crucial, not only for screening and diagnosis but also for pregnancy management and prognosis.

Thyroid hormones directly affect the body’s metabolism. Under the influence of TSH, the stimulating hormone secreted by the pituitary gland, the thyroid gland secretes triiodothyronine (T3) and tetraiodothyronine (T4). In their active form (free triiodothyronine (FT3) and FT4), these hormones play a role in homeostasis and energy metabolism. They stimulate gluconeogenesis in the liver and enhance glucose uptake in the small intestine. In addition, these hormones also participate in promoting lipid breakdown (lipolysis) and free fatty acid metabolism, thereby regulating blood glucose and lipid levels [13], 20]. During pregnancy, thyroid hormone levels usually increase slightly to meet the higher metabolic demands [13], 20]. Therefore, changes or disturbances in thyroid function during pregnancy can significantly affect glucose and lipid balance, indirectly impacting the risk of developing gestational diabetes. Additionally, thyroid dysfunction can alter insulin sensitivity. In hyperthyroidism, elevated thyroid hormones increase metabolic activity and energy consumption. This can lead to accelerated insulin degradation and increased glucose production in the liver, but is accompanied by increased insulin sensitivity in peripheral tissues. Conversely, in hypothyroidism, decreased T4 levels or elevated TSH levels, and slowed metabolism, particularly subclinical hypothyroidism (slightly elevated TSH levels but normal FT4 levels), are associated with greater insulin resistance [10], 11]. Insulin resistance is a central factor in the pathogenesis of GDM, so thyroid dysfunction may contribute to an increased risk of developing the disease. Furthermore, some authors suggest that FT4 hormone has a significant impact on the development and maturation of pancreatic islet β-cells, thus hypothyroidism is associated with insulin resistance, leading to an increased risk of diabetes and gestational diabetes [9], [10], [11, 21], 22]. However, the specific association between TSH and FT4 levels and the risk of gestational diabetes remains highly variable across studies [9], 11], 17], 22]. This inconsistency may be due to factors such as race, geographic location, genetics, living conditions, diagnostic criteria, and differences in the reference ranges of thyroid hormone tests used during pregnancy. In Vietnam, screening and assessment of thyroid function during pregnancy are still limited and not routinely or mandatorily performed in pregnant women. To date, no study in Vietnam has evaluated the longitudinal association between changes in TSH and FT4 hormone levels in early pregnancy and gestational diabetes. Based on this fact, we conducted this study to assess TSH and FT4 levels in early pregnancy as an early prognostic indicator of gestational diabetes risk, and to propose the application value of these tests in early screening for gestational diabetes in the community.

## Materials and methods

### Study subjects

This study was conducted on pregnant women aged 18–45 years carrying a singleton pregnancy conceived naturally, who visited Da Nang Hospital for Women and Children for prenatal care. Eligible participants were those indicated for TSH and FT4 quantification during the first trimester (from 9 to 13 weeks) and glucose tolerance testing during the second trimester (from 24 to 28 weeks). Inclusion in the study required the availability of complete research information and data.

Participants were excluded if they conceived via assisted reproductive technologies (IUI, IVF) or presented with acute illnesses such as infections, hepatic failure, or renal failure. The study also excluded pregnant women with a documented history of thyroid disease including hypothyroidism, hyperthyroidism (e.g., Graves’ disease), thyroid nodules, or cysts; or those who had received prior medical (thyroid hormones or antithyroid drugs) or surgical treatment for thyroid disorders, regardless of their current treatment status. Exclusion was based on clinical history rather than a specific TSH threshold to ensure the inclusion of women with physiological first-trimester thyroid variations.

Furthermore, women were excluded from the study if they met any of the following criteria: (1) a known pre-pregnancy history of diabetes mellitus (type 1 or type 2); (2) a diagnosis of overt diabetes in the first trimester, defined as fasting plasma glucose (FPG) ≥7.0 mmol/L (126 mg/dL), random glucose ≥11.1 mmol/L with hyperglycemic symptoms, or HbA1c≥6.5 %; or (3) suspected gestational diabetes or intermediate hyperglycemia detected in the first trimester (FPG≥5.1 mmol/L and/or HbA_1c_≥5.7 %) [23].

### Research methodology

A retrospective cohort study design was employed between December 2023 and March 2025. The study population comprised all eligible pregnant women visiting the hospital. Clinical data, including physical examination findings, thyroid function tests, glucose tolerance outcomes, and other relevant research indicators, were extracted using a standardized data collection form.

Gestational diabetes mellitus was diagnosed in accordance with the 2024 guidelines of the Vietnam Ministry of Health [23]. Participants underwent a 75 g, 2-h oral glucose tolerance test (OGTT) between 24 and 28 weeks of gestation. A diagnosis of GDM was confirmed if one or more plasma glucose values met or exceeded the following thresholds: fasting (G0): ≥5.1 mmol/L (92 mg/dL); 1-h (G1): ≥10.0 mmol/L (180 mg/dL); 2-h (G2): ≥8.5 mmol/L (153 mg/dL).

Research variables were collected based on maternal characteristics and biochemical markers. General characteristics included maternal age, pre-pregnancy Body Mass Index (BMI), parity, history of obstetric complications, and personal or family history of diabetes. Biochemical variables included serum TSH, FT4, and fasting blood glucose levels measured during the first trimester (<13 weeks), as well as OGTT results (G0, G1, G2) obtained in the second trimester (24–28 weeks). Serum TSH and FT4 concentrations were quantified using the Cobas 6000 analyzer (Roche Diagnostics) based on the electrochemiluminescence immunoassay (ECLIA) principle. The assays utilized standard commercial reagent kits (Elecsys TSH and Elecsys FT4 II) supplied by the manufacturer. The measurement process involved a two-point calibration strategy to ensure accuracy. Analytical reliability was maintained through strict adherence to internal quality control (IQC) and external quality assessment (EQA) procedures, in compliance with the Criteria for Evaluating the Quality of Medical Laboratories (issued under Decision No. 2429/QD-BYT by the Vietnam Ministry of Health).

Data analysis was conducted using STATA software, version 17.0. Descriptive statistics were utilized to characterize the study population, with categorical variables expressed as frequencies and percentages (n, %). For continuous variables, the normality of data distribution was assessed using the Shapiro-Wilk test. Consequently, variables such as maternal age, pre-pregnancy body mass index (BMI), and serum TSH and FT4 concentrations, which exhibited non-normal distributions (p<0.05), were summarized as medians and interquartile ranges (IQR). Comparative analysis of TSH and FT4 concentrations between the GDM and non-GDM groups was performed using the non-parametric Mann–Whitney U test. The predictive value of TSH and FT4 was assessed using receiver operating characteristic (ROC) curve analysis, reporting the area under the curve (AUC) with 95 % confidence intervals (95 % CI). To identify factors associated with GDM, univariate analysis was first performed on independent variables, including maternal characteristics and thyroid function markers. Subsequently, a multivariate logistic regression analysis was conducted to adjust for confounding factors and determine independent predictors. The multivariate model was constructed based on clinical relevance and established literature, regardless of statistical significance in the univariate analysis. Accordingly, the model included thyroid function parameters (FT4, TSH) and key covariates: maternal age, pre-pregnancy BMI, parity (number of previous pregnancies), family history of diabetes, and history of adverse obstetric outcomes. Results were expressed as adjusted odds ratios (aOR). A p-value <0.05 was considered statistically significant in the final model.

The study was approved by the Biomedical Ethics Committee of Da Nang University of Medical Technology and Pharmacy (Approval Certificate No. 61/CT-HDDD), and permission to collect data was obtained from Da Nang Hospital for Women and Children. All participant data were anonymized to ensure confidentiality.

## Results

A total of 272 pregnant women meeting the inclusion criteria were included in the final analysis. Table 1 summarizes the demographic, clinical, and biochemical characteristics of the study cohort. The maternal age ranged from 20 to 41 years, with a median age of 29 years (IQR: 26–33). The vast majority of participants (84.6 %) were under 35 years of age, while those aged 35 years or older accounted for 15.4 %. Regarding nutritional status, 8.1 % of women were classified as overweight or obese prior to pregnancy (BMI≥23). In terms of obstetric history, 130 women (47.8 %) were multiparous, and 25 (9.2 %) reported a history of obstetric abnormalities. The prevalence of other specific risk factors, including a personal or family history of diabetes mellitus or prior gestational diabetes, was low (<3 %). The overall prevalence of GDM diagnosed between 24 and 28 weeks of gestation was 13.6 % (37 cases). Regarding thyroid function profiles in the first trimester, the median TSH concentration was 0.86 mIU/L (IQR: 0.34–1.83 mIU/L), and the median FT4 concentration was 1.15 ng/dL (IQR: 1.04–1.27 ng/dL).

**Table 1:** General characteristics of the study subjects (n=272).

Characteristics	Median (IQR), n (%)
Maternal age, years	29 (26–33)
Age group	
<35	230 (84.6)
≥35	42 (15.4)
Pre-pregnancy BMI, kg/m^2^	20.7 (19.6–21.9)
BMI level	
<23	250 (91.9)
≥23	22 (8.1)
Number of previous pregnancies (parity)	
0	142 (52.2)
≥1	130 (47.8)
History of abnormal obstetrics	
No	247 (90.8)
Yes	25 (9.2)
History of diabetes or gestational diabetes	
No	265 (97.4)
Yes	7 (2.6)
Family history of diabetes	
No	264 (97.1)
Yes	8 (2.9)
Gestational diabetes (diagnosed at 24–28 weeks)	
No	235 (86.4)
Yes	37 (13.6)
TSH, mIU/L	0.86 (0.34–1.83)
FT4, ng/dL	1.15 (1.04–1.27)

**Figure 1:**
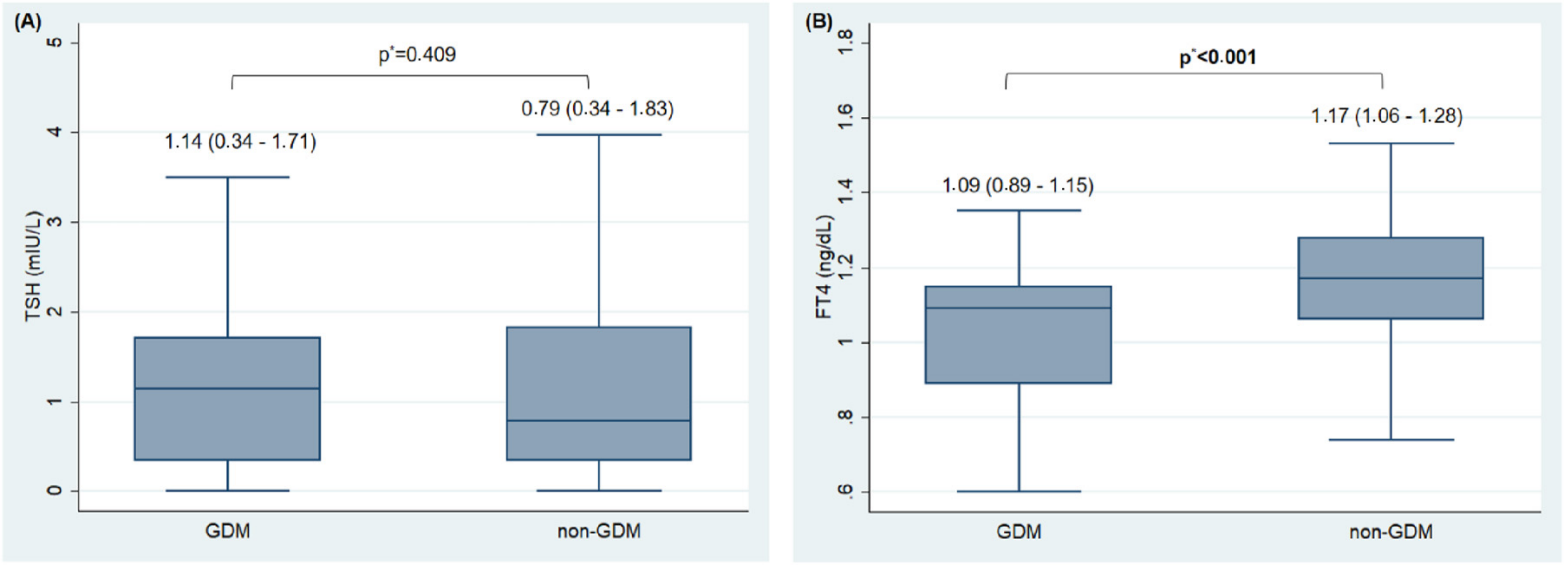
Comparison of TSH (A) and FT4 (B) concentrations in the first trimester of pregnancy in pregnant women with gestational diabetes (n=37) and without gestational diabetes (n=235), *Mann–Whitney U test.

Analysis of first-trimester thyroid profiles revealed no statistically significant difference in serum TSH concentrations between the GDM and non-GDM groups (p>0.05). In contrast, serum FT4 levels were significantly lower in the GDM group compared to the non-GDM group (p<0.001). Specifically, the median FT4 concentration in the GDM group was 1.09 ng/dL (IQR: 0.89–1.15), whereas the non-GDM group exhibited a higher median value of 1.17 ng/dL (IQR: 1.06–1.28) (Figure 1).

**Figure 2:**
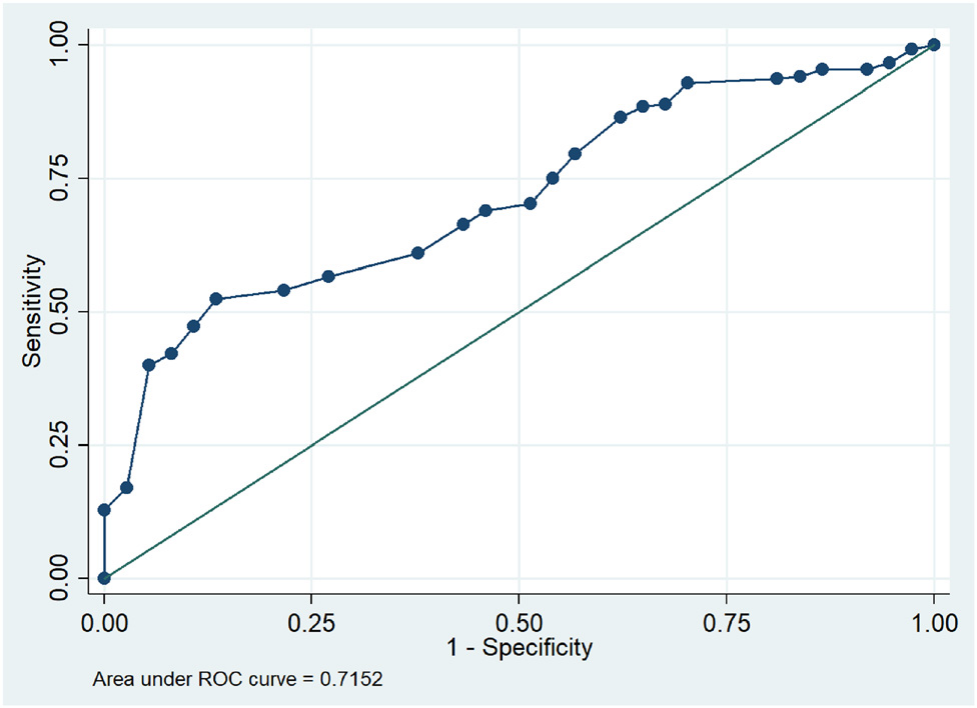
Predictive value of FT4 for gestational diabetes mellitus.

Receiver operating characteristic (ROC) curve analysis was performed to evaluate the predictive utility of thyroid function markers for GDM. An optimal cut-off value for FT4 concentration was identified at ≤1.17 ng/dL, demonstrating moderate predictive accuracy with an Area Under the Curve (AUC) of 0.715 (95 % CI: 0.636–0.794). At this threshold, sensitivity was 86.5 % and specificity was 52.3 % (Table 2, Figure 2). In contrast, TSH concentration showed limited predictive value; at a cut-off of 2.25 mIU/L, the AUC was 0.471 (95 % CI: 0.370–0.753), with a sensitivity of 89.2 % and a specificity of 16.2 % (Table 2).

**Table 2:** Values of TSH and FT4 in the first trimester in predicting GDM.

	Cut-off point	Sensitivity, %	Specificity, %	AUC (95 % CI)
TSH, mIU/L	≥2.25	89.2	16.2	0.471 (0.370–0.753)
FT4, ng/dL	≤1.17	86.5	52.3	0.715 (0.636–0.794)

Logistic regression analysis identified specific risk factors associated with the development of gestational diabetes mellitus. Notably, an FT4 concentration of ≤1.17 ng/dL was identified as an independent predictor of GDM, with an odds ratio of 5.84 (95 % CI: 2.13–16.00, p=0.001). Additionally, a pre-pregnancy BMI≥23 kg/m^2^ was significantly associated with an increased risk of the condition (OR=3.19, 95 % CI: 1.12–9.13, p=0.030) (Table 3).

**Table 3:** Relationship between TSH and FT4 levels in the first trimester of pregnancy and risk factors for gestational diabetes.

Characteristics	Gestational diabetes					
	n	%	aOR (95 % CI)	p_a_^a^	bOR (95 % CI)	p_b_^a^
TSH≥2.25 mIU/L	33	14.3	1.54 (0.50–6.33)	0.478	1.88 (0.59–5.96)	0.282
FT4≤1.17 ng/dL	32	21.2	6.24 (2.29–21.10)	**<0.001**	5.84 (2.13–16.00)	**0.001**
Age≥35	8	19.0	1.63 (0.59–4.05)	0.263	1.51 (0.59–3.89)	0.387
BMI≥23	7	31.8	3.48 (1.09–10.08)	**0.009**	3.19 (1.12–9.13)	**0.030**
Number of previous pregnancies (parity) ≥1	18	13.8	2.20 (0.66–6.29)	0.112	0.76 (0.34–1.69)	0.498
History of abnormal obstetrics	6	24.0	3.13 (0.28–19.96)	0.194	2.61 (0.86–7.94)	0.092
History of gestational diabetes mellitus	2	28.6	4.41 (0.65–23.67)	0.067	2.67 (0.41–17.57)	0.307
Family history of diabetes mellitus	3	37.5	2.20 (0.66–6.29)	0.112	2.58 (0.54–12.34)	0.237

^a^Logistic regression – a: univariate model – b: multivariate model (TSH≥2.25 mIU/L; FT4≤1.17 ng/dL; age≥35; BMI≥23; number of previous pregnancies; history of obstetric abnormalities; history of gestational diabetes; family history of diabetes). Bold values indicate p<0.05.

## Discussion

The association between thyroid dysfunction and diabetes mellitus (DM) has been well-established in numerous prior investigations [9], 17], 21], 24]. During pregnancy, thyroid dysfunction represents one of the most prevalent endocrine disorders [13], [24], [25], [26]; according to current American Thyroid Association (ATA) guidelines, its diagnosis relies principally on alterations in serum TSH and FT4 levels [13]. Concurrently, GDM, a distinct form of diabetes arising during gestation, is becoming increasingly common, driven by factors such as advanced maternal age, pre-pregnancy obesity, and sedentary lifestyles. Glucose homeostasis in pregnancy is intricately regulated by placental hormones, including human chorionic gonadotropin (hCG), estrogen, and insulin [2], 13], 27]. Emerging evidence suggests that perturbations in thyroid hormone levels, specifically elevated or suppressed TSH and diminished FT4, may serve as potential risk factors for the development of GDM [22], [28], [29], [30]. However, the magnitude and consistency of this relationship remain equivocal, likely attributable to variations in genetic background, nutritional status, lifestyle factors, and diagnostic assay standardization across different study populations.

In this study, no significant disparity in TSH levels was observed between the GDM and non-GDM groups. Conversely, FT4 levels were significantly lower in the GDM cohort, suggesting that FT4 may serve as an indicator of basal metabolic alterations occurring during early gestation. These findings align with numerous international investigations reporting diminished FT4 levels in populations at high risk for GDM [31], 32]. From a pathophysiological perspective, FT4 plays a pivotal role in regulating energy metabolism, directly influencing pancreatic β-cell function and peripheral insulin sensitivity. In the context of GDM, insulin resistance and glucose dysregulation may occur concomitantly with subtle perturbations in the hypothalamic-pituitary-thyroid axis. This implies that a reduction in FT4, even in the absence of significant TSH deviations, may reflect early endocrine dysfunction. However, it remains to be elucidated whether this decline in FT4 possesses distinct prognostic and pathophysiological significance or merely represents a physiological adaptation to early pregnancy. Consequently, further research integrating multiple metabolic markers and stratified risk analyses is warranted to fully characterize the role of FT4 in the pathogenesis of gestational diabetes.

In contrast to FT4, first-trimester TSH levels did not exhibit a statistically significant difference between the GDM and non-GDM groups in our study. This finding is largely attributable to the physiological influence of human chorionic gonadotropin (hCG), which must be considered when interpreting thyroid function in early pregnancy. During the first trimester, circulating hCG levels peak and exert a potent thyrotropic effect due to the structural homology between the alpha subunits of hCG and TSH. This homology allows hCG to cross-react with the TSH receptor on thyroid follicular cells, stimulating FT4 production and leading to a compensatory feedback suppression of pituitary TSH. This physiological suppression narrows the variability of TSH among euthyroid women, likely rendering it a less sensitive marker than FT4 for detecting subtle metabolic dysregulation in this specific gestational window. Furthermore, it is important to note that our study excluded women with a known history of thyroid disease to minimize confounding factors. While this design strengthens the internal validity of the study by isolating the risk in previously healthy women, it may limit the direct extrapolation of these findings to the unselected general population, where pre-existing thyroid disorders are present.

An outstanding finding of this study is the identification of a cut-off point FT4 threshold of ≤1.17 ng/dL, which demonstrated significant predictive utility for gestational diabetes mellitus. At this cut-off, the assay exhibited high sensitivity (86.5 %) and moderate specificity (52.3 %), with an Area Under the Curve (AUC) of 0.715 (95 % CI: 0.636–0.794), underscoring the value of low FT4 levels in early risk stratification (first trimester of pregnancy). Of particular clinical relevance is the high negative predictive value (95.9 %), which suggests that FT4 levels >1.17 ng/dL may effectively rule out imminent risk of GDM. While the relatively low specificity may lead to a higher rate of false-positive risk identification, in screening strategies, high sensitivity and NPV are prioritized to ensure that at-risk individuals are not overlooked. The high negative predictive value (NPV) is of particular clinical significance, indicating that women with FT4 levels >1.17 ng/dL have a lower probability of developing GDM, paving the way for effective exclusion of a low-risk subgroup in early pregnancy. Conversely, a low FT4 level (≤1.17 ng/dL) serves as an early warning signal for risk stratification. Clinically, this does not imply that all women with low FT4 require an immediate oral glucose tolerance test (OGTT); instead, they should be prioritized for early nutritional counseling, lifestyle modification, and closer metabolic monitoring. An early OGTT may be indicated if low FT4 is accompanied by other established risk factors, ensuring timely intervention without overburdening the healthcare system. Furthermore, multivariate regression analysis confirmed that both FT4≤1.17 ng/dL and pre-pregnancy overweight/obesity (BMI≥23 kg/m^2^) are independent risk factors for GDM. This result aligns with many international studies indicating that hypothyroxinemia in the first trimester is significantly associated with an elevated risk of developing gestational diabetes [21].

However, some previous studies present different data regarding the predictive value of thyroid hormones. Huang (2024) showed that while a TSH level <1.24 mIU/L remained significantly associated with gestational diabetes mellitus after adjustment for confounders, FT4 did not retain statistical significance (p=0.777) [9]. The discrepancies may be attributable to heterogeneity in population demographics, iodine supply levels, thyroid autoimmunity status, or differences in FT4 quantification techniques. Furthermore, the inconsistencies between previous studies may also arise from differences in nutrition, iodine deficiency rates, testing technology, as well as the inconsistent use of TSH-FT4 reference ranges [17]. This underscores the imperative to establish region-specific reference ranges and diagnostic cut-offs. Consequently, the FT4 threshold of ≤1.17 ng/dL identified in the present study should be interpreted as specifically applicable to the pregnant population in Danang, Vietnam, and areas with similar characteristics.

Notably, some classic risk factors for gestational diabetes mellitus, such as advanced maternal age (≥35 years) and a history of abnormal obstetric complications or diabetes, did not retain statistical significance following correlation analysis model. This may be due to several limitations, including the relatively modest sample size and the single-center design. Additionally, the reliance on retrospective data may have introduced potential inaccuracies regarding medical history, and the analysis was further limited by the absence of specific metabolic and endocrine covariates, such as urinary iodine concentration and thyroid autoantibodies (anti-TPO). However, despite these limitations and the inability to control for all potential confounding factors, this study provides robust evidence that low FT4 in the first trimester is significantly associated with an elevated risk of GDM. These findings reinforce the utility of FT4 as an early predictive biomarker, when integrated with clinical parameters such as BMI, maternal age and other clinical factors, to optimize risk stratification models and facilitate timely intervention strategies.

### Limitations

Several limitations of this study should be acknowledged. First, the sample size (n=272) is relatively modest compared to large-scale population-based studies. This is partly attributable to the rigorous exclusion criteria applied to ensure a homogeneous study population free of pre-existing thyroid or metabolic disorders. Specifically, our cohort was demographically skewed toward younger reproductive ages, with women aged ≥35 years comprising only 15.4 % (42/272) of the sample. Consequently, the lack of statistical significance for advanced maternal age in our model likely reflects a Type II error due to the low frequency of events in this subgroup, rather than contradicting the established literature regarding age as a major risk factor. Similarly, certain covariates (e.g., family history of diabetes or abnormal obstetric history) had low observational counts (n<10), which may result in wider confidence intervals and reduced stability of statistical estimates; these secondary associations should therefore be interpreted with caution.

Second, regarding the assay performance, it is acknowledged that the specificity of the FT4 cut-off at 1.17 ng/dL was relatively low (52.3 %), potentially leading to a higher rate of false-positive risk identification. However, in screening strategies, high sensitivity (86.5 %) and a high negative predictive value (NPV, 95.9 %) are often prioritized to minimize the likelihood of overlooking individuals at potential risk. The high NPV suggests that FT4 levels>1.17 ng/dL in early pregnancy may assist in the early identification of a low-risk subgroup regarding GDM development. Clinically, a low FT4 level should serve as an early warning signal for risk stratification, prioritizing these women for early lifestyle counseling and closer metabolic monitoring, rather than mandating immediate oral glucose tolerance test (OGTT) for all cases.

Third, due to the retrospective nature of the study, our analysis was restricted to TSH and FT4 concentrations, which are the standard markers used for routine antenatal screening at our institution. Data regarding Free Triiodothyronine (FT3), thyroid autoantibodies (TPOAb, TgAb), and maternal iodine status were not available. This is an important limitation, as these factors can significantly influence both FT4 levels and overall thyroid function during pregnancy. Consequently, the lack of these unmeasured variables might have confounded the observed associations and could partially explain the moderate predictive performance of FT4 in our cohort.

Finally, as a single-center study conducted at a tertiary hospital, the findings may reflect the specific characteristics of the local population and may not be fully generalizable to other regions with different iodine status or ethnic backgrounds. Future multi-center prospective studies incorporating a comprehensive thyroid profile and larger sample sizes are warranted to validate these findings and establish population-specific reference ranges.

## Conclusions

The present study suggests that first-trimester FT4 levels are independently associated with the risk of gestational diabetes mellitus, potentially reflecting underlying metabolic dysregulation in early pregnancy. While heterogeneity in global literature may be attributable to variations in iodine status, thyroid autoimmunity, and population-specific genetic characteristics, our findings support the potential involvement of thyroid function in glucose homeostasis. Clinically, the integration of FT4 assessment with established risk factors, such as maternal age, BMI, and obstetric history, may enhance the identification of at-risk pregnancies. This multimodal approach facilitates early clinical oversight, thereby enabling the implementation of timely, individualized monitoring and preventive interventions. However, given the moderate predictive performance (AUC 0.715), FT4 should be viewed as a potentially useful marker for risk stratification rather than a definitive screening tool. Further large-scale prospective studies are warranted to validate these diagnostic thresholds and determine the feasibility of routine FT4 screening in prenatal care.
